# Spatial response of water level and quality shows more significant heterogeneity during dry seasons in large river-connected lakes

**DOI:** 10.1038/s41598-024-59129-w

**Published:** 2024-04-10

**Authors:** Yingze Yin, Rui Xia, Xiaoyu Liu, Yan Chen, Jinxi Song, Jinghui Dou

**Affiliations:** 1 Shaanxi Key Laboratory of Earth Surface System and Environmental Carrying Capacity, College of Urban and Environmental Sciences, Northwest University, Xi’an, 710127 China; 2 State Key Laboratory of Environmental Criteria and Risk Assessment, Chinese Research Academy of Environmental Sciences, Beijing, 100012 China; 3 National Joint Research Center for Ecological Conservation and High-Quality Development of the Yellow River Basin, Beijing, 100012 China; 4 National Engineering Laboratory for Lake Pollution Control and Ecological Restoration, Chinese Research Academy of Environmental Sciences, Beijing, 100012 China; 5 Chongqing Institute of Green and Intelligent Technology, Chinese Academy of Sciences, Chongqing, 400714 China

**Keywords:** Spatial response, Water level, Water quality, River-connected lake, Wavelet correlation (WTC), Self-organizing map (SOM), Hydrology, Limnology, Environmental monitoring, Geochemistry

## Abstract

The spatial response mechanism of hydrology and water quality of large river-connected lakes is very complicated. In this study, we developed a spatial response analysis method that couples wavelet correlation analysis (WTC) with self-organizing maps (SOM), revealing the spatial response and variation of water level and water quality in Poyang Lake, China's largest river-connected lake, over the past decade. The results show that: (1) there was significant spatial heterogeneity in water level and quality during the dry seasons (2010–2018) compared to other hydrological stages. (2) We identified a more pronounced difference in response of water level and quality between northern and southern parts of Poyang Lake. As the distance increases from the northern lake outlet, the impact of rising water levels on water quality deterioration intensified during the dry seasons. (3) The complex spatial heterogeneity of water level and quality response in the dry seasons is primarily influenced by water level fluctuations from the northern region and the cumulative pollutant entering the lake from the south, which particularly leads to the reversal of the response in the central area of Poyang Lake. The results of this study can contribute to scientific decision-making regarding water environment zoning management in large river-connected lakes amidst complex environment conditions.

## Introduction

The hydrological rhythm and water quality changes in the confluence area of river–lake systems exhibit a high degree of complexity and uncertainty^1,2^. Affected by the backward flow and jacking of the mainstream of rivers, the response of lake water level and water quality shows typical non-stationary characteristics^3^. Due to factors like basin topography, hydrological situation and complicated river–lake relationship^4^, spatial differences in the hydrological environment of river-connected lakes can also be substantial. During flood seasons, fresh water entering the lake dilutes pollutants, while nutrients from the main water channel are transported to the nearshore area, promoting primary production^5,6^. However, when floodwaters recede, organic particles and nutrients are carried back to the main channel, worsening the environment of river-connected lakes^7^. In low-connected hydrological states, the hydrodynamic force in some areas may not be enough to naturally remove excess nutrients, leading to increasing spatial differences in water quality within the lakes^8^. Well-known river-connected lakes like Usumacinta^9^, Tonle Sap Lake^10^, and Poyang Lake^11^ all experience poorer water quality and greater spatial heterogeneity during the dry seasons compared to the wet seasons. This issue poses a challenge for interdisciplinary research combining hydrology and environmental studies.

In recent years, natural hydrological patterns of many lakes have been altered for various purposes, such as flood control, power generation, maintaining shipping and ecological water demand. These interventions have resulted in more frequent extreme water level variations and unknown ecological and environmental impacts^12^. However, research on the changing relationship between water level and water quality in highly dynamic lakes and the mechanisms behind their complex spatial heterogeneity is still lacked. Poyang Lake, as a globally recognized river-connected lake, is not only China’s largest freshwater lake but also one of the most remarkable lakes in Northeast Asia. Throughout the year, Poyang Lake experiences a seasonal water level fluctuation exceeding 10 m, presenting a unique hydrological phenomenon where the lake transforms into a river form and then reverts back to a lake form^13^. However, since the twenty-first century, the dry seasons of Poyang Lake have been advancing and prolonging gradually. The annual average water area and volume have decreased, and the degradation of water quality and ecological environment persisted^14,15^. The understanding of the response mechanism of hydrological variations and water quality changes in the lake remains unclear, especially considering the complex spatial scale of hydrological and water environment data. Unveiling the spatial distribution pattern of the water environment’s response under complex hydrological conditions poses a substantial challenge^16^.

Studying the relationship between water level and water quality in lakes is crucial due to the nonlinear and cumulative nature of changes in the hydrological environment^17^. Previous studies have examined this relationship, but the findings have been inconsistent. For instance, studies on river-connected lakes like Poyang Lake and Dongting Lake have shown a negative correlation between water level and the concentration of nitrogen and phosphorus nutrients^5,11,18^. However, these studies have overlooked the potential nonlinear changes within this relationship. To address this issue, wavelet correlation analysis (WTC) has emerged as a valuable tool for identifying nonlinear temporal patterns in hydrological and environmental responses^19^. WTC analyses have indicated that the correlation between water level and nutrient concentration in rivers and lakes is nonlinear^20^. In particular, the study on large river-connected lake has shown that the hydrological conditions and water quality do not always follow a stable one-way response mechanism^3^. The substantial increase in hydrological fluctuation amplitude can significantly impact the relationship between water level and water quality. However, although WTC provides insights into the nonlinear aspect, it does not explain spatial heterogeneity. Therefore, it is necessary to explore the spatial distribution differences and evolutionary processes of the response relationship in a scientific and visually appealing manner.

Therefore, our study aims to uncover the spatial patterns and variations of nonlinear response between water level and quality in Poyang Lake, the largest river-connected lake in China. Our specific objectives include: (1) Identify the spatial heterogeneity of water level and water quality in Poyang Lake from 2010 to 2018 and determine the most significant hydrological stage and its spatial characteristic. (2) Quantitatively describe the intensity and relative phase of the correlation between water level and water quality at 13 sampling sites in the lake using WTC. We will then use self-organizing map (SOM) to cluster the samples based on these two correlation variables. (3) Divide the lake into typical areas based on the spatial differentiation patterns of the nonlinear response clusters. Additionally, we will discuss the key factors that may contribute to these spatial differences.

## Materials and methods

### Study area

Poyang Lake, located in Jiangxi Province in China, is the largest freshwater lake in the country and stands out for its natural connection with the Yangtze River^13^. It receives water primarily from five rivers: Gan, Fu, Xin, Rao, and Xiu River, accounting for about 89% of the total inflow^21^. After being controlled and stored, the lake water is discharged into the Yangtze River through a narrow northern outlet. Poyang Lake plays a crucial role in China, contributing over 18% of the total runoff of the Yangtze River and supporting approximately 40% of the country’s population. The surrounding area is characterized by farmland, woodland, and wetland habitats.

Affected by the subtropical monsoon, the average annual precipitation in Poyang Lake ranges from 1450 to 1550 mm, with the majority falling between May and September, accounting for about 60% of the total rainfall. The complex interaction of irregular precipitation distribution, inflowing rivers, and the Yangtze River leads to water level fluctuations in Poyang Lake, ranging from below 8 m to above 20 m. The lake itself also exhibits unique topographical features, including high-flow channels, gently undulating flood plains, bays, and islands^22^.

### Data collection and water quality assessment method

**Figure 1:**
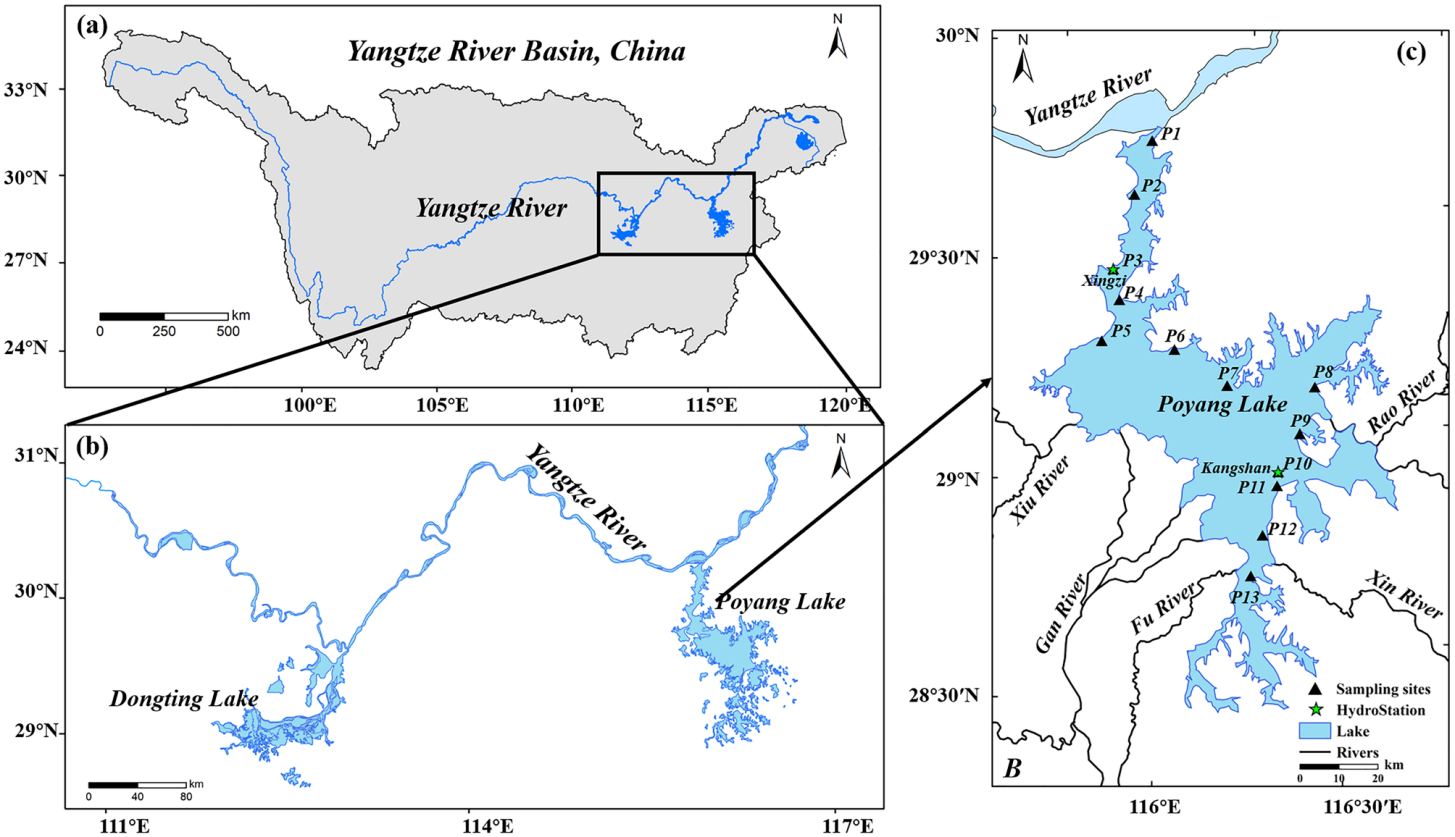
Schematic view of the study area. (**a**) The Yangtze River Basin, China; (**b**) water system in middle reach of Yangtze (**c**) Poyang Lake tributary river inlets, the lake outlet and sampling sites—created using ArcGIS v10.2^23^.

In this study, the monthly water quality data of 13 points from 2010 to 2018 were obtained from Jiangxi Ecological Environment Monitoring Center (Fig. 1). To comprehensively assess the changes in water quality, according to the relevant requirements of the "Surface Water Environmental Quality Evaluation Method (Trial)", we selected permanganate index, chemical oxygen demand, five-day biochemical oxygen demand, ammonia nitrogen, and total phosphorus as the water quality evaluation indicators. These parameters were used to calculate the comprehensive water quality index (CWQI), with Class III water quality established by the Environmental Quality Standard for Surface Water of China (No.: GB3838-2002) serving as the benchmark (the higher the CWQI value, the worse the water quality)^24^. The calculation formula is as follows:

1 $$CWQI=1/n\sum_{i=1}^{n}{c}_{i}/{c}_{0}$$

where C_i_ is the measured value of the ith parameter, C_0_ is the standard value of the ith parameter, and n is the number of parameters. In this paper, C_0_(COD_mn_) = 6 mg/L, C_0_ (COD) = 20 mg/L, C_0_ (BOD_5_) = 4 mg/L, C_0_ (NH^3^-N) = 1 mg/L, and C_0_ (TP) = 0.05 mg/L.

In addition, the water level data of Xingzi Station and Kangshan Station are obtained through Hydrological Yearbook of Jiangxi Province, China (http://tjj.jiangxi.gov.cn/).

### Method for identifying spatial response of water level and water quality

**Figure 2:**
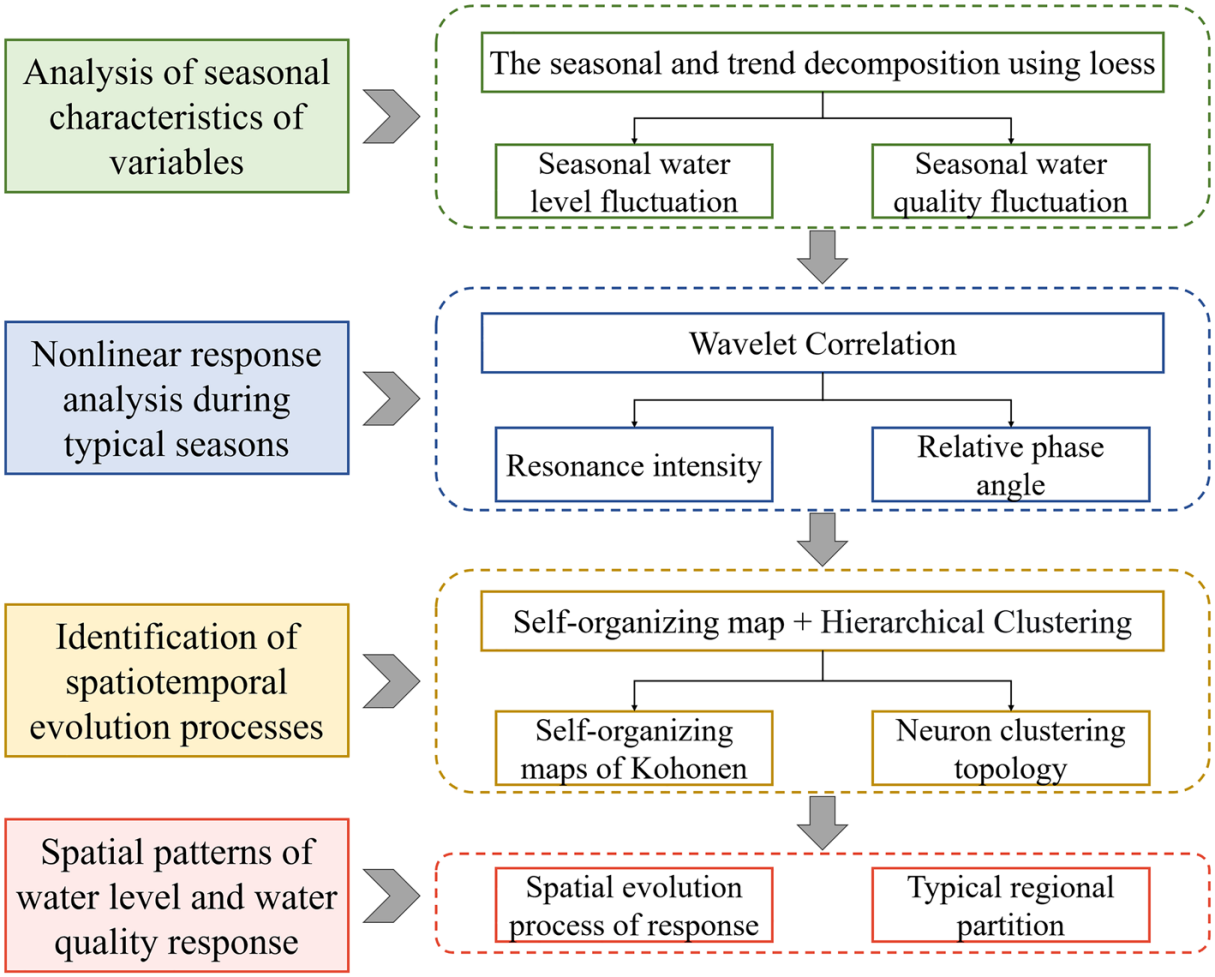
Approach for identifying spatial response of water level and water quality.

In this study, we employ mathematical and statistical methodologies to analyze water level and quality data from 13 sampling sites in Poyang Lake (2010–2018) (Fig. 2). Firstly, to account for the typical seasonal variation of Poyang Lake, we use the seasonal and trend decomposition using loess (STL) method to characterize seasonal dynamics by separating long-term trends from seasonal variations. Significance of trend changes is determined using the Mann–Kendall (M–K) and Pettitt tests. Following this, we identify the hydrological stage with the most spatial heterogeneity. Secondly, we use WTC analysis to capture the nonlinear relationship between water level fluctuations and water quality changes. To explain spatial dimensions, we incorporate SOM analysis, integrating hierarchical clustering outcomes for temporal and spatial distribution characteristics. Ultimately, we propose a methodology centered on WTC-SOM for identifying the spatial distribution of the water level and water quality response in large river-connected lakes.

#### The seasonal and trend decomposition using Loess (STL)

The Seasonal and Trend decomposition using Loess (STL) method is a statistical approach for analyzing time series data. In this method, a robust loess weighted regression is used as a smoother to fit a weighted polynomial regression to the observed data over time^25^. The weights assigned to each data point decrease as the distance from the point to the observation window increases. By applying the loess smoother, the time series ${Y}_{t}$ of time T(t) can be decomposed into three components:

2 $${Y}_{t}={T}_{t}+{S}_{t}+{R}_{t}\left(t=\mathrm{1,2},3,\dots ,T\right)$$

where ${Y}_{t}$ is the long-term trend, ${S}_{t}$ is the seasonality and ${R}_{t}$ is the residual^26^. The trend component represents the overall long-term change in the time series, whereas the seasonal component captures the periodic fluctuations that occur within each season^27^. Trend and seasonal components extracted from hydrological and water quality data can be regarded as relatively independent components, and random interference can be avoided. In this study, the “stats” package in R is utilized to implement the STL method.

#### Wavelet correlation analysis (WTC)

Wavelet correlation analysis is widely recognized as the optimal approach for examining the correlation between nonlinear time series^19^. This sophisticated technique enables the assessment of both the magnitude and phase relationship of two time series in the time–frequency domain by discerning frequency bands and time intervals^28^. For two time series x and y, WTC can be defined as:

3 $${R}_{n}^{2}\left(s\right)={\left|S({s}^{-1}{W}_{n}^{XY}(s))\right|}^{2}/S({s}^{-1}{\left|{W}_{n}^{X}(s)\right|}^{2})\cdot S({s}^{-1}{\left|{W}_{n}^{Y}(s)\right|}^{2})$$

where ${R}_{n}^{2}\left(s\right)$ represents wavelet correlation, and its value range is 0–1; The closer the value is to 1, the greater the correlation between the two sequences^29^. S represents the smoothing operator of scale and time domain.

4 $$S\left(W\right)={S}_{scale}({S}_{time}({W}_{n}^{X}(s)))$$

where ${S}_{scale}$ is smooth along the scale axis and ${S}_{time}$ is smooth along the time axis. The “WaveletComp” package in R is utilized to implement the Wavelet Coherence (WTC) methodology in this study.

#### Self-organizing map (SOM)

Self-organizing map (SOM) is an unsupervised artificial neural network technique developed by Kohonen in 1982. It allows for the representation of high-dimensional data into a lower-dimensional format, often displayed as a two-dimensional grid known as a “feature diagram”^30^. SOM is commonly used for visualization and pattern classification to identify data features effectively^31^. The size of the map, which refers to the number of output neurons, is crucial in implementing SOM^32^. Researchers have discovered that the optimal number of neurons typically approximates 5√n, where n represents the number of samples^33^. Experimental testing has determined that a neuron architecture of 7*9 achieves the highest recognition ability and captures the most information in this study. The "Kohonen" package in R is utilized in this study to implement SOM^34^.

Since SOM cannot automatically determine the optimal number of clusters^35^, hierarchical clustering analysis (HCA) is used in this research to divide the samples. The node weight vector of SOM serves as the input for clustering analysis, and clustering boundaries are depicted on the graph to identify clusters^36^. The optimal number of clusters is determined based on the minimum Davies-Bouldin index, which assesses the compactness and separation of the clusters^37,38^. After clustering the SOM node graph, the Shapiro–Wilk test is performed to evaluate the normality of the dataset. As the dataset does not follow a normal distribution, the Kruskal–Wallis test is employed to assess the differences in all variables between clusters.

## Results

### Spatial characteristics of water level and water quality in different seasons

**Figure 3:**
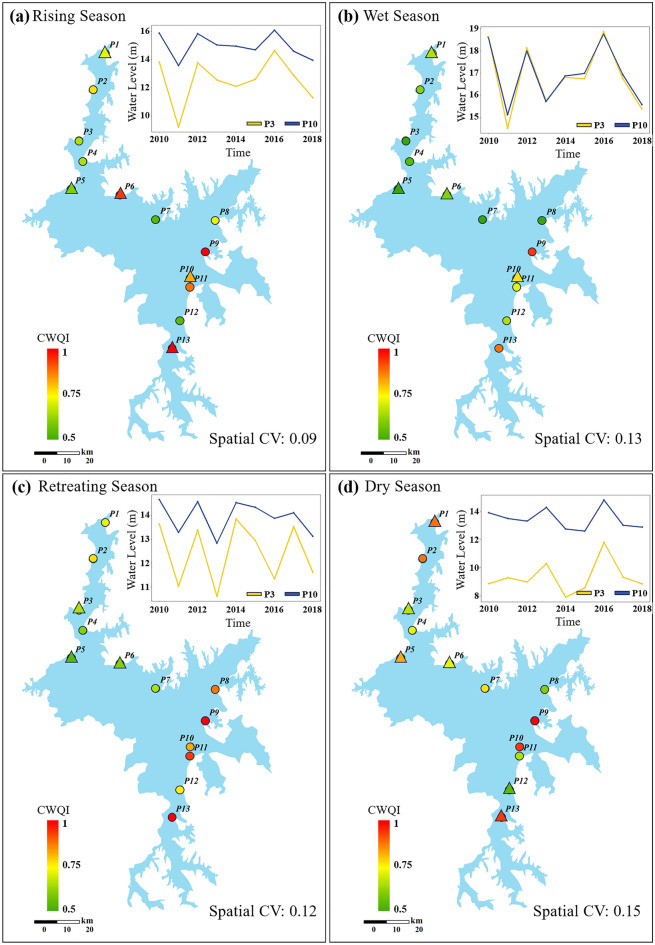
Spatial distribution of multi-year comprehensive water quality index (CWQI) and water level. Circular icons represent no significant change in water quality, while triangles indicate a significant upward trend. The coefficient of variation (CV) value denotes the extent of water quality dispersion among the 13 points—created using ArcGIS v10.2^23^.

According to the hydrological process, Poyang Lake goes through four distinct hydrological stages: rising season (Mar–May), wet season (Jun–Aug), retreating season (Sep–Nov), and dry season (Dec-Feb)^39^. Figure 3 shows the monthly average water levels at Xingzi (p3) and Kangshan (p10) in Poyang Lake and the multi-year average water quality of 13 sampling sites from 2010 to 2018. During the wet seasons, there is minimal spatial difference in lake water levels. However, during the other hydrological stages, the water level in the northern lake area (p3) is consistently lower compared to the southern area (p10). The disparity in water level between these two points is most pronounced during the dry seasons, with average water levels spanning 9.35 m and 13.44 m respectively over multiple years. Furthermore, the spatial coefficient of variation (CV) of the multi-year average water quality during the dry seasons is 0.15, indicating the highest degree of spatial heterogeneity in water quality during this period.

**Figure 4:**
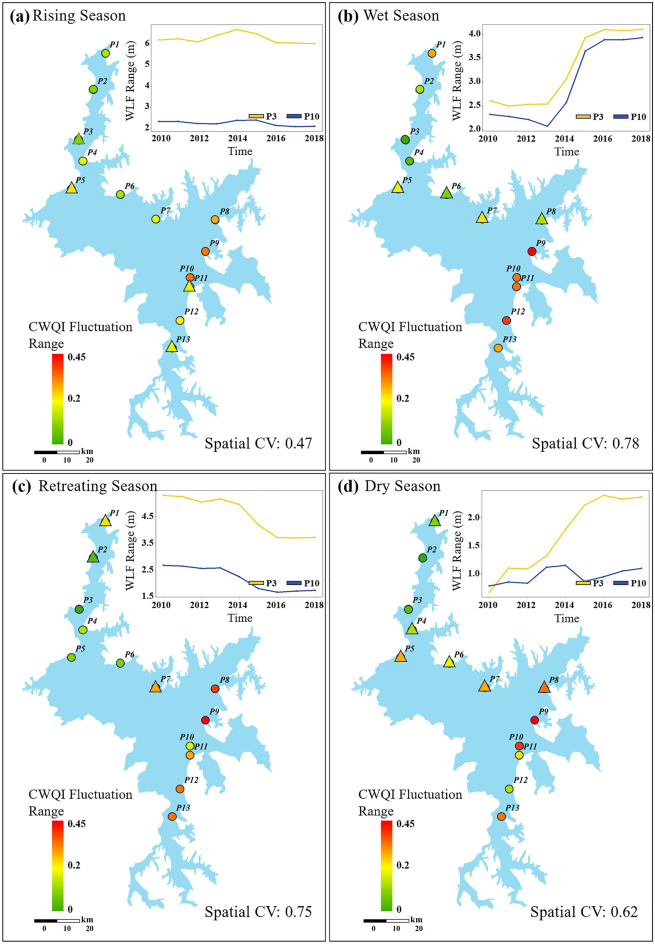
Spatial distribution of seasonal fluctuation range of multi-year average comprehensive water quality index (CWQI) and water levels (WLF). Circular icons represent no significant change in water quality, while triangles indicate a significant upward trend. The coefficient of variation (CV) value denotes the extent of water quality fluctuation range dispersion among the 13 points—created using ArcGIS v10.2^23^.

Figure 4 shows the distribution of water level and water quality fluctuations range at different points, which the fluctuation range is calculated as the difference between the maximum and minimum values of the seasonal items, decomposed by STL, in a given year/season. In the four hydrological stages, the water level fluctuation curve for p3 consistently surpasses that of p10, indicating that the northern region of the lake experiences larger fluctuations compared to the southern region. During the dry seasons, there is a notable spatial difference in water level fluctuations. The water level fluctuation at p10 remains relatively constant at around 0.89 m throughout the study period, while at p3, the average fluctuation is 1.7 m, with a significant increase over time and experienced a sudden change in 2014 (Pettit, P < 0.05). In other stages, the water level fluctuations at both points remain relatively consistent. The spatial CV value for water quality fluctuations is smallest during the wet seasons, measuring 0.47. However, there is a considerable spatial difference during the other seasons, especially during the dry seasons where around 46% of the points in the lake show a significant upward trend in water quality fluctuation range (M–K, P < 0.05). Therefore, our focus lies on establishing a spatial response for water level and water quality during the dry seasons. Due to limitations in data integrity, we use the Thiessen Polygon method to establish the relationship between water quality data from 13 points within the lake and water level data from Xingzi and Kangshan stations.

### Identification the response between water level and water quality during dry seasons

Based on the authors' research^3^, a quantitative analysis using WTC identified nonlinear responses between water level and water quality at 13 points. These responses primarily included resonance intensity and relative phase angle. Table 1 presents the findings, indicating that the average response intensity during the dry seasons in Poyang Lake remained consistently above 0.85, with a small CV value. This suggests a strong correlation between water level fluctuations and water quality changes during this period. Although the average phase angle did not vary significantly in the dry seasons (M–K, P > 0.1), the CV value showed a significant increase (M–K, P < 0.05). This indicates a substantial increase in the spatial heterogeneity of the responses. Consequently, different areas of the lake are evolving in different directions.

**Table 1:** The average, standard deviation (SD), and coefficient of variation (CV) for resonance intensity and relative phase angle between water level fluctuation and water quality change in dry seasons.

	Resonance intensity			Relative phase angle		
	Average	SD	CV	Average	SD	CV
2010	0.88	0.21	0.24	171.67	92.33	0.54
2011	0.87	0.24	0.28	203.27	86.87	0.43
2012	0.85	0.18	0.21	195.65	74.92	0.38
2013	0.82	0.15	0.18	169.49	100.38	0.59
2014	0.86	0.13	0.15	170.19	95.44	0.56
2015	0.81	0.20	0.24	199.77	101.31	0.51
2016	0.74	0.26	0.35	172.37	120.96	0.70
2017	0.87	0.12	0.14	179.14	132.74	0.74
2018	0.94	0.06	0.07	204.04	136.07	0.67
Average	0.85	0.17	0.21	185.07	104.56	0.57

**Figure 5:**
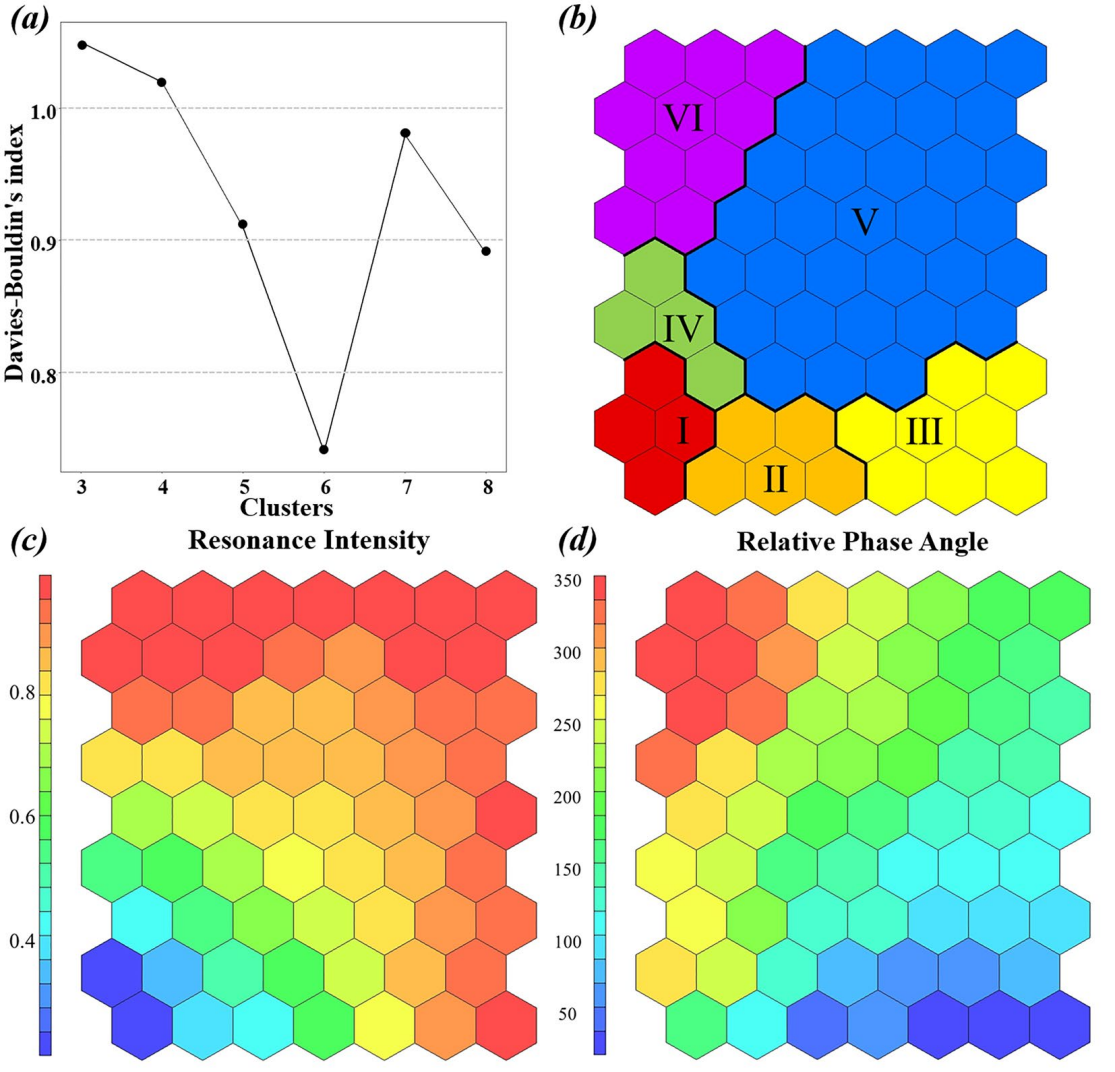
Self‐organizing maps of Kohonen: (**a**) Davies-Bouldin index plot. (**b**) map of samples, in which the samples were divided in 6 distinct groups (I–VI). (**c**,**d**) component planes for the variables analyzed in the SOM.

**Figure 6:**
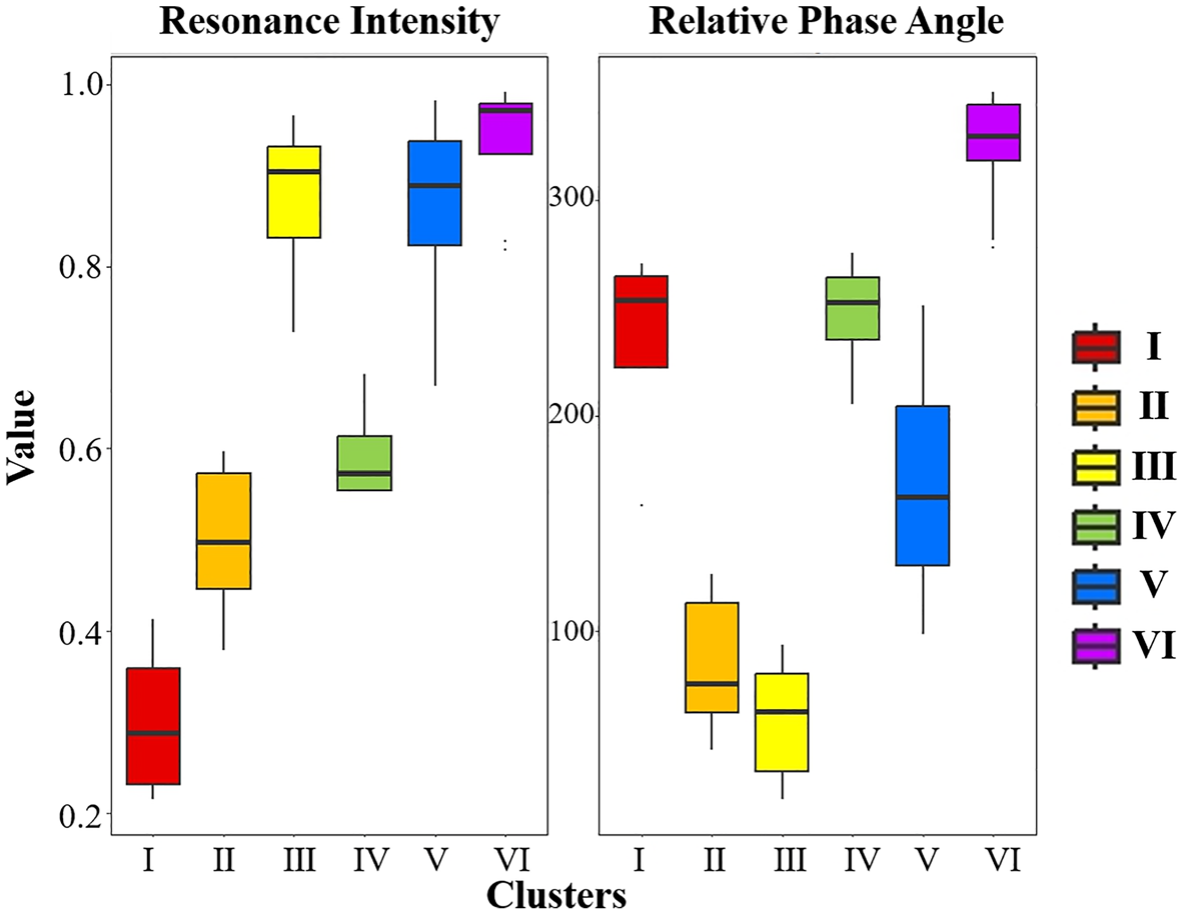
Box plots of the variables within each cluster.

Subsequently, the 117 sample data obtained from wavelet correlation analysis underwent further analysis using SOM. Figure 5a shows that the Davies-Bouldin index reached its minimum value when the trained SOM output neurons were hierarchically clustered into six categories (I-VI) (Fig. 5b). The clustering results were validated through the Kruskal–Wallis test (P < 0.01). Figure 5c,d display the component planes of each variable. Figure 6 displays the distribution of variables in each cluster. Clusters I, IV, and V show similar phase angles in the second and third quadrants (between 120° and 270°), indicating a negative correlation between water level and water quality at these sample points. However, there are noticeable differences in their response intensity. Cluster I represents a weak response, cluster IV represents an intermediate transition state, and cluster V represents a strong correlation. Most samples in clusters II and III have phase angles in the first quadrant (between 0° and 90°), suggesting a positive correlation. Cluster II can be categorized as a transitional state with a weak response, while cluster III exhibits a strong correlation. Cluster VI, on the other hand, has a phase angle in the fourth quadrant (between 270° and 360°), indicating a delay in the response between the two variables. This group shows a distinct positive phase relationship compared to the other clusters. It’s important to note that in wavelet correlation analysis, where we assume that water level fluctuations cause changes in water quality, caution is needed when interpreting phase angles exceeding 270°. Interpreting these results solely as a positive correlation would be insufficient.

### Spatial distribution of water level-water quality response during dry seasons

**Figure 7:**
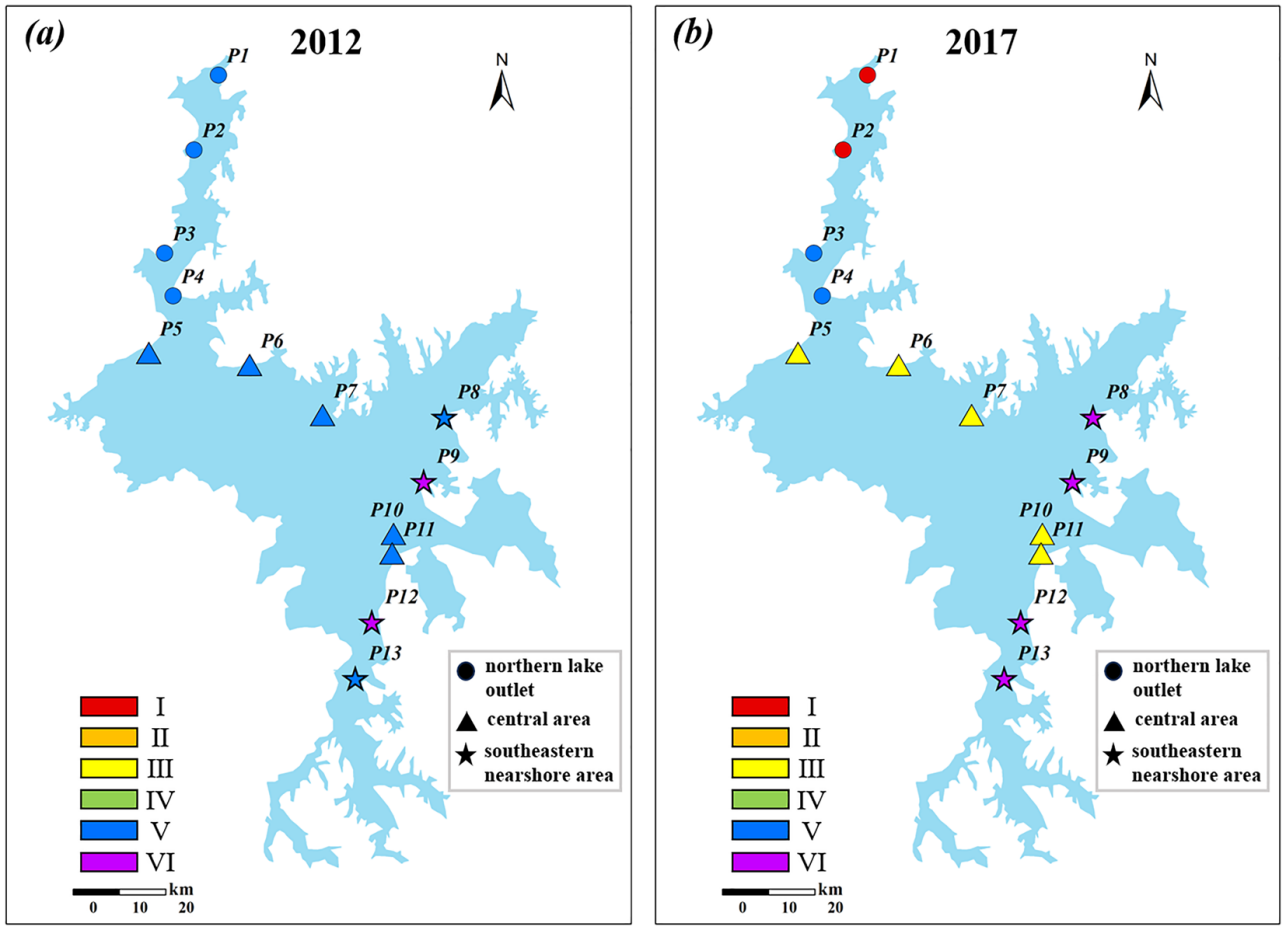
Spatial distribution of clusters in typical years: (**a**) shows 2012 and (**b**) shows 2017—created using ArcGIS v10.2^23^.

In Fig. 5b, each sample in the original data is assigned to the nearest output neuron, referred to as the best matching unit (BMU). This assignment ensures that each sample belongs to the same cluster as its corresponding BMU. Except for cluster V, the clusters have a relatively localized distribution. More detailed results can be found in Supplementary Fig. S1 online. Spatial distribution maps were created for the years 2012 and 2017, as shown in Fig. 7. Cluster V is widely distributed throughout the entire lake, especially during the period of 2010–2013. Since 2014, cluster III has been continuously spreading within the lake area (p5–11). Cluster VI mainly occupies the southeastern nearshore area and its distribution range is gradually expanding. At the same time, cluster I consistently appears in the northern lake outlet (p1,2).

**Figure 8:**
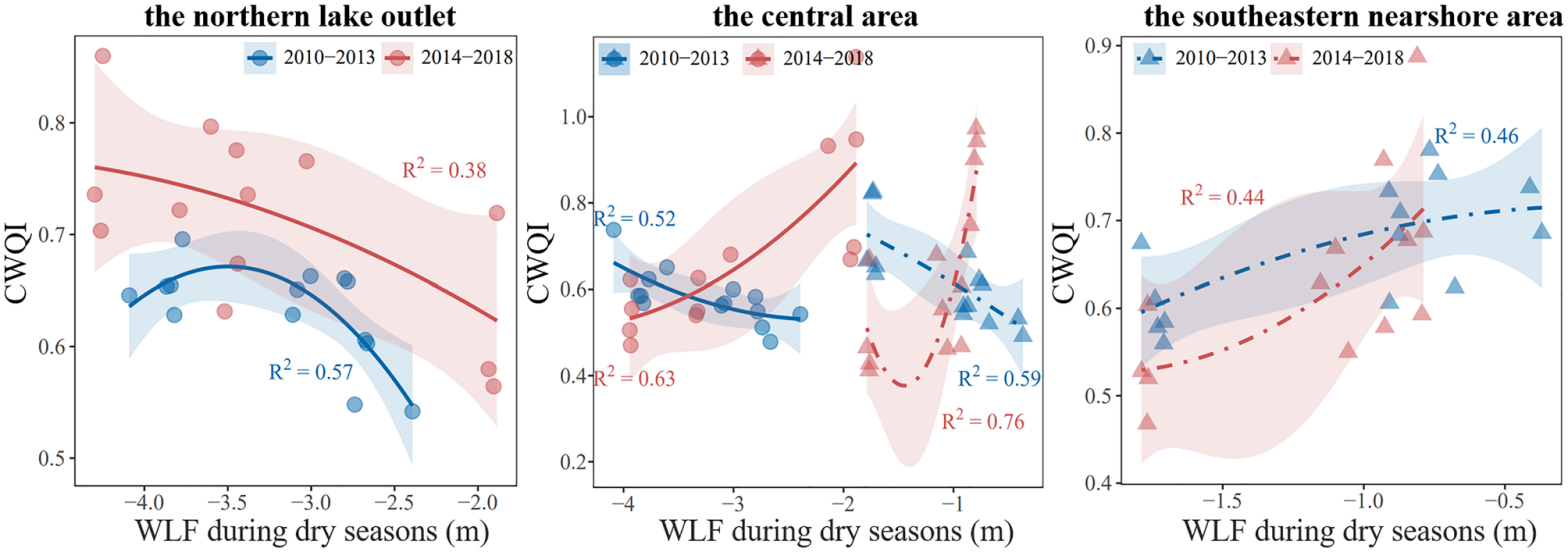
Schematic diagram of the changes in comprehensive water quality index (CWQI) during dry seasons in three representative areas in relation to water level fluctuations (WLF). Where circles represent the samples influenced by water level changes at Xingzi Station, while triangles represent the samples affected by water level changes at Kangshan Station.

Based on the spatial variation characteristics of the clustering results, the entire lake area can be categorized into three typical regions: the northern lake outlet, the center of the lake, and the southeastern nearshore area. Figure 8 shows that there is a negative correlation (cluster V) between water level and water quality in the northern lake outlet (p1–4), indicating that water quality improves as water level rises during the dry seasons. However, after 2014, a sudden increase in water level fluctuations weakened the correlation, as evidenced by the decrease in the R^2^ value of the fitting trend (cluster I). The central part of the lake (p5, 6, 7, 10 and 11) is the most sensitive to changes in water level. Around 2014, the relationship between water quality and rising water levels shifted from negative correlation (cluster V) to positive correlation (cluster III), suggesting a shift from initial improvement to deterioration. In the southeastern nearshore area, although water quality worsened with rising water levels, the low R^2^ value observed throughout the study period indicates that various complex factors beyond water level changes influence water quality in this particular region.

## Discussion

### Spatial heterogeneity of water level and water quality in large river-connected lakes during dry seasons

Our research uncovers that the spatial variation in water levels during dry seasons is highly significant. Compared with the average water level, the spatial heterogeneity of seasonal water level fluctuation amplitude is more prominent, specifically manifested in the water level fluctuation amplitude in northern river-connected area being significantly higher than that in the south, with a sudden increase occurred in 2014. This time coincides with the arrival of the smallest water area in Poyang Lake in nearly 40 years^15^. The Poyang Lake basin slopes from east to west and from south to north, and this natural topography of the lake results in a spatial pattern of water level distribution. This characteristic becomes particularly prominent during the dry seasons, when the water level continuously decreases and eventually recedes into the deeper channel. The water level changes are influenced by the gravitational gradient of the main trough, thereby amplifying the difference between the north and south^40,41^.

In addition, compared to the tributaries flowing into Poyang Lake, the interaction between the Yangtze River and the lake has a greater impact on the water level changes in the dry seasons^42^. Due to the increasing water storage capacity of reservoirs in the upper and middle reaches of the Yangtze River during autumn and winter, combined with the scouring and erosion along the middle and lower reaches of the river, the water level of the Yangtze River decreases significantly^15^. This leads to an increase in the water level difference between the rivers and the lake, resulting in a greater emptying effect of the main stream of the Yangtze River on the water level of Poyang Lake^43,44^. However, the influence of changes in the water level of the Yangtze River on Poyang Lake decreases with increasing distance from the lake’s exit^45^. Our research also shows that the change in the river–lake relationship primarily affects the Xingzi point, while having no significant influence on the Kangshan station in the southern lake area.

Moreover, our research indicates that the spatial heterogeneity of lake water quality is pronounced during the dry seasons. In particular, in the northern region where the water level is low and the fluctuation amplitude increases significantly, there is a considerable upward trend in water quality and its fluctuation amplitude. This suggests that water level change has a substantial impact on water quality. However, due to the distinct spatial differences and sudden variations in water level within Poyang Lake during the dry seasons, the response of water level to water quality may vary temporally or spatially. Therefore, we propose the WTC-SOM coupling method to comprehensively depict the detailed changing trend of this relationship.

### Nonlinear spatial response identification approach using WTC and SOM analysis

Our study agrees that there is a complex and changing relationship between water level and water quality in river-connected lakes. To fully comprehend this relationship, it is crucial to understand its nonlinear nature. We also need to examine how it varies across different areas, especially in large lakes that are heavily influenced by human activities. To tackle these research objectives, we have introduced a methodology that combines the WTC and SOM analysis. This integration allows us to identify and analyze spatial distribution patterns and temporal evolution of the non-linear relationship between water level and water quality.

Traditional correlation analysis methods such as Pearson, Spearman, and grey correlation may not capture the intricate changes in correlation over time accurately. Hence, we employed the WTC analysis to decompose time series data into multiple components for different time periods. This approach simplifies the complex data and provides a quantitative representation of the non-linear characteristics of the water level-water quality relationship^46^. While the WTC analysis helps us understand how the relationship varies over time, it does not directly reveal the spatial distribution of this relationship in large river-connected lakes with significant spatial variations. To address this challenge, we introduce the SOM method. Self-organizing map (SOM), a clustering technique based on artificial neural networks, have been increasingly employed to classify water environment data and recognize spatiotemporal distribution patterns^31,47,48^. It is particularly useful for uncovering local relationships that may be hidden by global exploration^49,50^. However, SOM has limitations in explaining nonlinear correlations among input variables. Previous studies have used Spearman or visual inspection of attribute map color distribution to qualitatively analyze variable correlations^4,51^.

In our analysis, we emphasize the complementary relationship between WTC and SOM. WTC measures nonlinear correlation changes, while SOM enhances the analysis by incorporating spatial dimensions. By using WTC, we found that the relationship between water level and water quality at certain points shows changes in resonance intensity and relative phase over time. This confirms that the relationship between water level and water quality in large river-connected lakes has noticeable nonlinear characteristics, which were often overlooked in previous studies. Additionally, SOM is an effective tool for identifying and visually representing spatio-temporal samples with similar response characteristics. This simplifies the explanation of complex multi-point spatio-temporal issues and helps us uncover local spatial heterogeneity in the water level-water quality response at 13 monitoring points within the lake area. These findings emphasize the importance of considering spatial heterogeneity in the study of water environments in large lakes and provide valuable insights for further discussions of key influencing factors in sub-regional.

### Driving factors of spatial evolution in water level-water quality response in large river-connected lakes during dry seasons

Most studies suggest that the water level-water quality response in large river-connected lakes is influenced by both human activities and local environmental factors^11,52^. In shallow lakes, sediment resuspension is dominant during the dry seasons and serves as an important source of nutrients^10,53,54^. In areas with significant hydrological variation, prolonged periods of relative drought and severe water level fluctuations can accelerate the exposure and release of pollutants from sediments^55^. Furthermore, sudden influxes of colder water into the lake can cause the lower water layers to rise and mix with the surface water layer^56^. As a result, rapid changes in water flow can lead to a deterioration of water quality in certain parts of the lake (p5–7, Fig. 8b). However, the channel closer to the lake outlet exhibits narrower terrain and faster water flow^57^. Here, the short-term impact of water level disturbances on sediment release is partially offset, maintaining a negative correlation between water level and water quality. Although the water quality improvement resulting from water level increase remains largely unchanged, there is a noticeable weakening of the dilution effect on pollutants near the mouth of the lake (p1,2, Fig. 8a).

In regions heavily impacted by human activities, human runoff has increased pollutants in more developed areas, making them more sensitive to land use compared to natural areas^58^. Poyang Lake basin is important for commodity grain production in China, with a cultivated land area of 7659.29 km^2^, concentrated mainly in the southern area^59^. Previous studies have shown that phosphorus accumulation in the terrestrial system surrounding the Poyang Lake basin had reached critical levels as early as 2005^60,61^. When the nutrient retention capacity of the terrestrial system reaches its threshold, the lake contributes to its buffering capacity, with sediment becoming the primary destination for a significant portion of the pollutants^62^. As a result, the same water level fluctuations in certain middle areas of the lake have shifted the impact on water quality from improvement to deterioration (p10–11, Fig. 8b). Furthermore, the influence of sediment pollutant load on water quality follows a gradually weakening trend from the nearshore area to the lake outlet area^55^. Therefore, a weak positive correlation between water level and water quality consistently exists in the southeast nearshore areas (p8, 9, 12, 13, Fig. 8c). The sustained and strong impact on the deterioration of water quality is mainly caused by the accumulation of pollutant load resulting from human activities, rather than solely hydrological factors.

In conclusion, during the dry seasons, the deterioration of water quality due to rising water levels is more pronounced in regions farther from the northern lake outlet. This can be attributed to the fluctuation of water levels in the northern region and the increased influx of pollutants from the south. In the northern area, the sharp rise in water level fluctuations accelerates the release of pollutants trapped in sediments. However, the lake outlet’s unique geographical location facilitates the rapid outflow of pollutants. Consequently, while there is still a negative correlation between water level and water quality, the sharp hydrological changes weaken the positive impact of rising water levels on water quality improvement. In the central area of the lake, which is affected by rapid hydrological changes and the accumulation of pollutants, we observed a reversal from a strong negative correlation to a strong positive correlation between water level and water quality. This shift indicates a change from water quality improvement to aggravation in response to rising water levels during the dry seasons. Moving closer to the southeast, in the nearshore area, we consistently observed a weak positive correlation between water level and water quality. Here, the continuous and significant deterioration of water quality is primarily driven by the high cumulative load of pollutants, which becomes the dominant factor replacing water level fluctuations.

In the future, the construction of reservoirs in the upper and middle reaches of the Yangtze River may further intensify the shift between dry and wet processes in Poyang Lake, leading to a continuous decline in water levels^22^. Given that Jiangxi Province is a major agricultural region, there is unlikely to be an immediate reduction in fertilizer consumption^61^, and the accumulated pollutant load is expected to remain persistently high. Our research underscores the evolving relationship between water level and water quality in different regions of Poyang Lake under multiple environmental pressures. To comprehend and forecast the evolving trends in water environments within large river-connected lake systems, it is imperative to closely examine the distinct hydrological responses across various regions.

## Conclusions

In this study, we proposed a spatial response identification method using wavelet correlation analysis (WTC) and self-organizing map (SOM) to uncover the spatial nonlinear response and differentiation patterns of water level variation and water quality in Poyang Lake, the largest river-connected lake in China. Our findings showed that significant spatial heterogeneity in water level and water quality is more significant during the dry seasons. Poyang Lake can be divided into three regions based on the water level-water quality response characteristics: the northern lake outlet, the center of the lake, and the southeast nearshore area. As the distance from the northern outlet increases, the deterioration impact of water level rise on water quality during the dry seasons gradually intensified. This spatial heterogeneity is caused by the combined influence of rapid hydrological changes from the north and the accumulation of high pollutant loads in the south. The application of the WTC-SOM coupling method in this study provided a scientific foundation for understanding the nonlinear response of lake water level and water quality under complex hydrological conditions. This research deepened our comprehension of the spatial distribution and changes in the long-term water environment system of lakes and contributes valuable insights for water environment zoning management in river-connected lakes.

### Supplementary Information

Supplementary Figure S1.

## Data Availability

The data that support the findings of this study are available from [Jiangxi Ecological Environment Monitoring Center] but restrictions apply to the availability of these data, which were used under license for the current study, and so are not publicly available. Data are however available from the authors upon reasonable request and with permission of [Jiangxi Ecological Environment Monitoring Center].
