# Supplementary material for: Spatial response of water level and quality shows more significant heterogeneity during dry seasons in large river-connected lakes
Source: Sci Rep. 2024 Apr 10;14:8373. doi: 10.1038/s41598-024-59129-w (PMC11006923; doi:10.1038/s41598-024-59129-w)
Supplement: Supplementary file 1 — Supplementary Figure S1. [file 41598_2024_59129_MOESM1_ESM.docx]

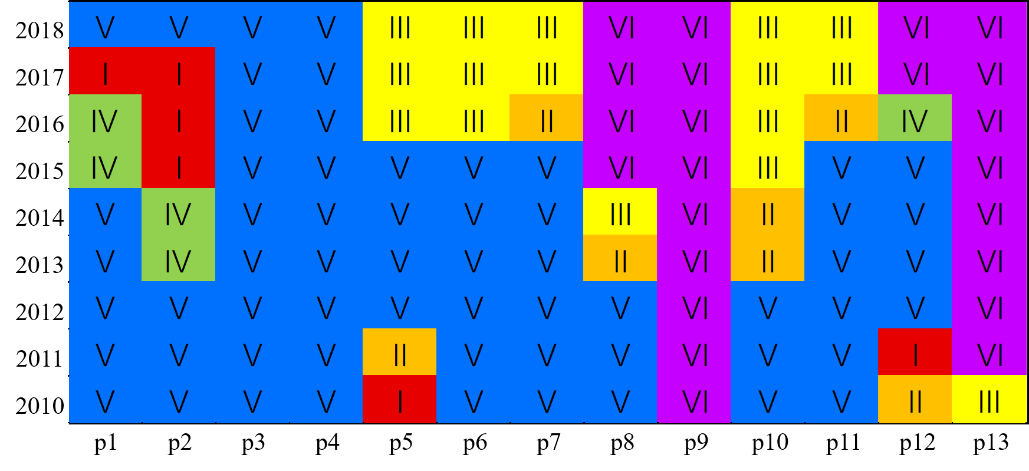


**Supplementary Fig. S1.** Temporal and spatial distribution of six clusters, with the corresponding cluster index number displayed on each sample for easy interpretation.
